# Safety and efficacy of bone marrow-derived cells therapy on cardiomyopathy: a meta-analysis

**DOI:** 10.1186/s13287-019-1238-5

**Published:** 2019-05-20

**Authors:** Chao Wang, Jingzhao Li, Boya Zhang, Yongjian Li

**Affiliations:** Department of Cardiology, Tianjin Nankai Hospital, No. 6 Changjiang Road, Nankai District, Tianjin China

**Keywords:** Bone marrow cells, Cardiomyopathy, Ventricular remodeling, Ventricular function

## Abstract

**Background:**

Controversial results still existed on the clinical utility of bone marrow-derived cells (BMCs) for cardiomyopathy (CMP). This study aims to reveal the true power of this promising approach by synthesizing all the available data on this subject matter.

**Methods:**

Twenty studies including 1418 patients were identified from systematic search. Weighted mean differences for changes in left ventricular ejection fraction (LVEF), left ventricular end-diastolic volume (LVEDV), left ventricular end-systolic volume (LVESV), 6-min walk distance, and NYHA functional class were estimated with a random-effects model. Major adverse cardiovascular event (MACE), rehospitalization, all-cause mortality, and patients’ quality of life were also calculated.

**Results:**

Compared with the control group, BMC therapy resulted in greater LVEF (3.72%, 95% CI 2.31 to 5.13, *P* < 0.0001), 6-min walk distance (53.16, 95% CI 25.17 to 81.10, *P* = 0.0002), NYHA functional class (− 0.48, 95% CI − 0.65 to − 0.31, *P* < 0.0001), and smaller LVESV (− 16.79, 95% CI − 27.21 to − 6.38, *P* = 0.002). BMC treatment significantly reduced the mortality rate and improved patients’ quality of life. No significant difference was found between the BMCs and control group in LVEDV, MACE, and rehospitalization rate. However, the outcomes showed a clear trend in favor of the BMC group. Subgroup analysis showed that LVEF improved greater in a subgroup of intracoronary infusion, BMSC, or higher cell dose.

**Conclusion:**

The results of the current meta-analysis suggest that BMC treatment for CMP is safe and feasible. This therapy was associated with persistent improvements in LV function, LV remodeling, functional class, patients’ survival, and quality of life. Intracoronary infusion of high-dose (> 10^8^) BMSC might be a better therapeutic option for CMP patients. Further evidences are needed to verify our results.

## Introduction

Cardiomyopathy (CMP) is a group of diseases that affect the heart muscle. The common types of CMP are hypertrophic cardiomyopathy, dilated cardiomyopathy, and restrictive cardiomyopathy. Heart failure, most often develops as a result of CMP, is the leading cause of mortality worldwide. Many former treatments have been applied to cure CMP, including medical therapy, cardiac pacemaker implantation, and heart transplantation. However, these traditional therapies cannot fundamentally solve the problems of cardiomyocyte regeneration and cardiac function reconstruction, making the therapeutic effect of cardiomyopathy unsatisfactory [1, 2]. In recent studies, with the newly emerged stem cell therapy, myocardial regeneration and cardiac function reconstruction have become possible [3, 4].

Bone marrow-derived cells (BMCs), mainly consists of hematopoietic stem cells and mesenchymal stem cells, under appropriate conditions can differentiate into mesoderm and ectoderm tissues [5]. Because of its convenience and safety, and it is easy to culture in vitro and its autologous replantation, BMCs have become an important cell source for tissue engineering [6]. A number of studies have shown that BMC transplantation can be successful in homing and colonizing pathological myocardial tissue, derived into different cells which help enhance myocardial contractility, promote myocardial angiogenesis, and prevent adverse ventricular remodeling [7, 8]. It has been performed in the treatment of cardiac pain, myocardial infarction, heart failure, and other cardiac diseases.

Multiple clinical trials have shown that BMC transplantation is safe and feasible in the treatment of cardiomyopathy [9, 10], while others remain controversial. Some of the researches indicated that BMCs improve heart function, increase activity tolerance, and reduce malignant arrhythmia morbidity and mortality [11–13]. Other studies suggested that BMC transplantation does not prevent progression, nor reduce mortality. On the contrary, it has the potential to trigger complications such as arrhythmias, myocardial infarction, embolism, and tumor [14]. The inconsistency of these research findings may be on account of the small sample size, the cause of the cardiomyopathy, the condition of the disease, the type of cell use in the transplantation, the number and route of the cell transplantation, and the length of treatment and follow-up duration.

In order to evaluate the efficacy and safety of BMC transplantation in the treatment of cardiomyopathy more objectively and accurately, we have conducted this meta-analysis. By comparing and analyzing the result of the currently published researches related to BMC transplantation for cardiomyopathy, we aim to provide more reliable evidence for clinical use.

## Methods

### Data source and search strategy

A database search of PubMed and EMBASE was performed to retrieve relevant publications up to March 2019. Search terms were chosen to link stem cell therapy with cardiomyopathy and its treatment effect. The following search terms were used: “cardiomyopathy,” “non-ischemic cardiomyopathy,” “dilated cardiomyopathy,” “idiopathic cardiomyopathy,” “stem cell,” “bone marrow cells,” “mesenchymal stem cell.” We also hand searched the reference lists of identified articles, review, and editorial for additional studies. There was no restriction on studies in terms of year and language of publication.

### Study selection

Prospective randomized controlled trials (RCT) assessing the left ventricular functions and clinical outcomes in CMP patients treated with BMC transplantation were enrolled in our meta-analysis. Besides, the eligible studies need to fulfill the following criteria: (1) patients with diagnostic CMP receiving stem cell therapy, including bone marrow mononuclear cells (BMNC) and bone marrow mesenchymal stem cells (BMSC); (2) studies that had at least 1 month of follow-up; (3) studies provided proper functional outcomes such as left ventricular ejection fraction (LVEF), left ventricular end-diastolic volume (LVEDV), left ventricular end-systolic volume (LVESV), 6-min walk distance, New York Heart Association (NYHA) functional class, and exercise capacity or clinical outcomes regarding major adverse cardiovascular events (MACE), mortality rate, rehospitalization rate, and quality of life; (4) studies included a control group which did not receive cell therapy. We excluded articles which were reviews, editorial, and abstracts presented at a conference. Duplicate reports and ongoing or unpublished studies were also excluded.

### Data extraction and quality assessment

Investigators independently screened all titles and abstracts to identify whether the studies met our inclusion criteria and extracted relevant data using a standardized form. For each eligible study, we extracted information regarding characteristics of the study (first author, year of publication, study design, sample size), patients (age, gender, type of disease, baseline LVEF), and intervention (dosage, route of administration, imaging modality, timing of follow-up). The outcome measurement included changes in LVEF, LVEDV, LVESV, 6-min walk distance, NYHA functional class, VO_2_ peak, and patients’ quality of life. MACE, all-cause mortality, and rehospitalization rate were also collected. Outcomes measured by different modes of imaging included echocardiography (ECHO), cardiac magnetic resonance imaging (CMR), electrocardiogram (ECG), and single-photon emission-computed tomography (SPECT) were all extracted. When multiple measuring tools were used in one study, CMR and ECHO data were preferentially selected. Clinical trials with multiple publications with sequential follow-up duration or different outcomes were considered as one study.

Study quality was evaluated using the Cochrane Collaboration’s risk of bias tool [15]. Each study was judged by seven domains, concerning random sequence generation, allocation concealment, blinding of participants and personnel, blinding of outcome assessment, incomplete outcome data, selective reporting, and other sources of bias. For each domain, studies were rated as high (red), unclear (yellow), or low (green) risk of bias. Any disagreement was resolved by discussion.

### Data analysis

All analyses were performed using the Cochrane Collaboration Review Manager (version 5.3) software. Data extracted from each study were pooled by the use of the DerSimonian-Laird random-effects model. For continuous variables, we used the weighted mean difference (WMD) and 95% confidence intervals (CI) to estimate the treatment effects of each outcome. For dichotomous data, such as MACE rate and mortality rate, risk ratio (RR) with its 95% CI was calculated. Heterogeneity between studies was assessed by the *I*^2^ statistic (low 25–50%, intermediate 50–75%, high > 75%), and sensitivity analysis was conducted if significant heterogeneity was found (*I*^2^ > 50%) in any one of the outcomes. Sensitivity analysis was performed by removing one study at a time to reveal if one particular study could affect the overall result. Also, subgroup analysis stratifying studies according to follow-up duration, cell type, injection route, and dosage of BMCs was performed to discover the source of heterogeneity. Pooled outcomes were displayed using forest plots, and outcomes were considered as statistically significant if *P* value < 0.05.

## Results

### Search results

**Fig. 1:**
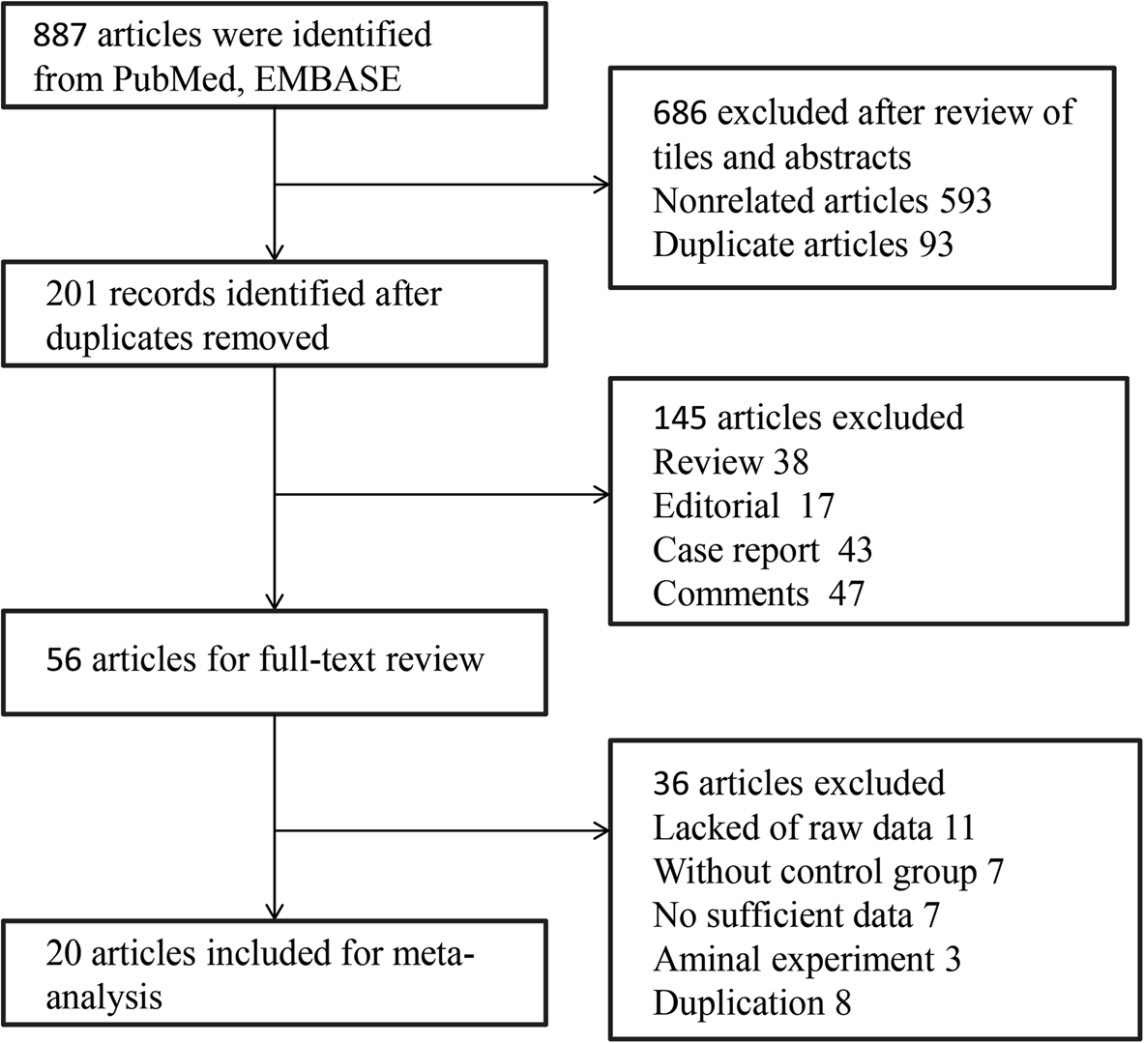
Flow diagram of the literature search in this meta-analysis

The systematic search identified 887 articles from PubMed and EMBASE. After reviewing all the titles and abstracts, 686 studies were excluded due to non-related topic and duplication, and 201 remained as potential candidates for our meta-analysis. We further excluded 145 studies since they did not meet our eligibility. Fifty-six studies were under full-text reviews and data extraction, of which 11 studies lacked of raw data, 7 articles did not include a control group, another 7 trials did not provide sufficient data for outcome analysis, 3 reports were an animal experiment, and 8 present repetitive data from an author with additional studies. Thus, the final 20 studies were included in our meta-analysis. Figure 1 presented the flowchart of a literature search.

### Study characteristics

The included 20 studies consisted of a total of 1418 patients, with 705 patients receiving BMC therapy and 713 patients served as control [14, 16–36]. We selected studies published between 2004 and 2017, which included the latest study of this subject matter. Studied size ranged from 20 to 258 patients, and the majority of trials used a 1:1 randomization scheme. Among the chosen studies, 8 trials included a BMSC treatment group, 14 used BMNC as seed cells, and 2 studies applied Ixmyelocel-T as their treatment cells. Patients’ age was varied widely among the included studies, ranging from 30 to 80. Male patients took up around 71% of the population. Detailed information regarding baseline LVEF, dosage of BMCs, and administration route was presented in Table 1. The imaging modality of the enrolled studies included CMR, ECHO, ECG, and SPECT, and data of cardiac parameters measured by the above appliances were considered equivalent. Table 1 summarized the characteristic of the included studies.

**Table 1:** Study characteristics

Author	Year	Group	*N*	Age	M/F	Disease	LVEF (%)	Dose	Route	Follow-up	Imaging
Bartolucci	2015	BMNC	12	58 ± 14	9/3	DCM	≤ 40	8.19 × 10^6^	IC	–	ECHO
		Control	11	57 ± 11	8/3						
Bartolucci	2017	BMSC	15	57.33 ± 10.05	12/3	DCM	≤ 40	1 × 10^6^	IV	12 months	ECHO
		Control	15	57.2 ± 11.64	14/1						
Chang	2010	BMNC	12	38.6 ± 9.8	16/8	DCM	–	2 × 10^8^	IM	6 months	ECHO
		Control	12								
Chen	2006	BMSC	22	59.3 ± 6.8	19/3	IDCM	< 40	5 × 10^6^	IC	12 months	SPECT
		Control	62	57.8 ± 7.2	21/2						
Chen	2008	BMNC	71	53 ± 15	44/17	DCM	< 45	–	IC	24 months	ECG
		Control	187	54 ± 13	136/51						
Hamshere	2015	BMNC	15	57.67 ± 12.32	10/5	DCM	< 40	2.16 × 10^8^	IC	12 months	CMR
		Control	14	54.87 ± 10.86	9/6						
Heldman	2014	BMNC	19	57.1 ± 10.6	18/1	IDCM	< 50	–	IM	12 months	CMR
		Control	10	61.3 ± 9.0	10/0						
Henry	2014	Ixmyelocel-T	39	64.7 ± 9	21/0	IDCM	< 30	2.95 × 10^8^	IM	12 months	ECG/SPECT
		Control	20	63.2 ± 12	9/0						
Martino	2015	BMNC	82	49.6 ± 11.1	53/25	NIDCM	< 35	2.36 × 10^8^	IC	12 months	ECHO
		Control	78	51 ± 1.11	60/22						
Nesteruk	2017	BMNC	114	66.7 ± 8.3	105/9	IDCM	< 45	3.9 × 10^6^	IM	4–14 years	ECHO
		Control	36	68.6 ± 5.78	22/4						
Patel	2016	Ixmyelocel-T	59	65.3 ± 8.49	55/3	IDCM	< 35	–	IM	12 months	ECHO
		Control	55	64.7 ± 9.94	45/6						
Perin	2004	BMNC	11	56.5 ± 7.8	9/2	DCM	< 40	–	IM	12 months	SPECT
		Control	9	58.9 ± 7.6	8/1						
Sant’Anna	2014	BMNC	15	48.3 ± 8.71	13/7	NIDCM	< 35	1.06 × 10^8^	IM	12 months	ECHO/CMR
		Control	9	51.6 ± 7.79	5/4						
Seth	2010	BMNC	41	45 ± 15	33/8	DCM	< 40	1.68 × 10^8^	IC	36 months	ECHO
		Control	40	49 ± 9	35/4						
Song	2008	BMSC	27			DCM	< 50	2 × 10^6^	IM	6 months	Ultrasound
		Control	25								
Vrtovec	2011	BMSC	28	52 ± 8	26/2	DCM	< 30	1.13 × 10^8^	IC	12 months	ECHO
		Control	28	54 ± 7	23/4						
Vrtovec	2013	BMSC	55	53 ± 8	45/10	DCM	< 30	–	IC	60 months	ECHO
		Control	55	55 ± 7	44/11						
Wang	2006	BMSC	12	54 ± 11.1	9/3	DCM	< 45	5.86 × 10^5^	IC	12 months	ECHO
		Control	12	58.4 ± 11	8/4						
Xiao	2017	BMNC	16	49.5 ± 11.6	9/7	DCM	< 40	5.1 × 10^8^	IC	3 months	SPECT
		BMSC	17	51.6 ± 12.2	12/5						
		Control	20	54.4 ± 11.6	14/6						
Yan	2012	BMSC	18	58 ± 9.9	12/6	DCM	< 40	1 × 10^8^	IC	12 months	SPECT/ECHO
		Control	20	58.2 ± 9.8	14/6						

*M/F* male/female, *LVEF* left ventricular ejection fraction, *IC* intracoronary, *IM* intramyocardial, *IV* intravenous, *QS* quality assessment, *BMNC* bone marrow mononuclear cells, *BMSC* bone mesenchymal stem cells, *DCM* dilated cardiomyopathy, *IDCM* ischemic dilated cardiomyopathy, *NIDCM* non-ischemia dilated cardiomyopathy, *ICM* ischemic cardiomyopathy, *CCC* Chagas cardiomyopathy, *CMR* cardiovascular magnetic resonance imaging, *ECHO* echocardiography, *ECG* electrocardiogram, *SPECT* single-photon emission-computed tomography

**Fig. 2:**
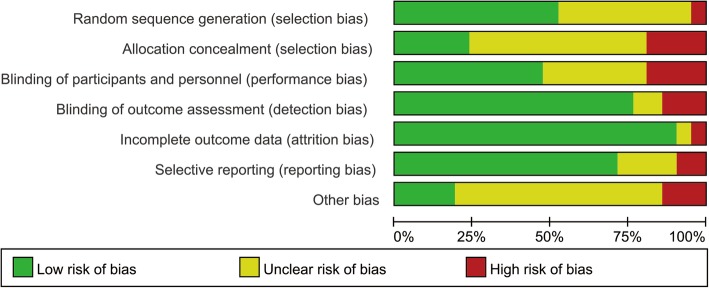
Risk of bias summary of 22 included studies

The methodological quality of the included trials was reckoned to be acceptable, as each domain was mostly ranked as low or unclear risk of bias. Low risk of bias mostly occurred in performance bias, detection bias, and attrition bias. Unclear risk of bias was mostly detected in selection bias, because many of the studies only mentioned that the study was RCT without describing the specific randomization and allocation concealment method. Summary of risk of bias analysis was presented in Fig. 2.

### Cardiac parameters of LV function

**Fig. 3:**
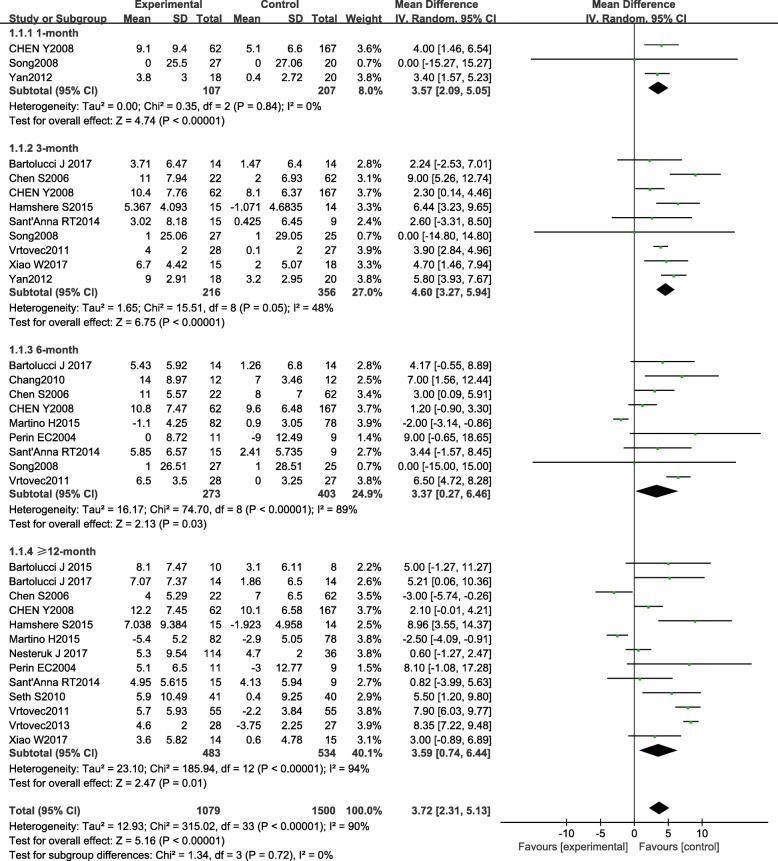
Forest plot of pooled left ventricular ejection fraction (LVEF) with bone marrow-derived cell treatment compared with the control group

The overall result revealed a significant improvement of LVEF by 3.72 (95% CI 2.31 to 5.13, *P* < 0.0001, *I*^2^ = 90%) (Fig. 3) in the BMC group compared with the control group. However, heterogeneity was high and many of the studies provided LVEF data at different follow-up period. It is less rigorous to only assess the combined effect. Thus, we estimated the BMC efficacy according to different time duration. Subgroup results showed that the beneficial effect of BMC therapy on LVEF exerted in 1-month (3.57, [95% CI 2.09 to 5.05], *P* < 0.0001, *I*^2^ = 0%), 3-month (4.60, [95% CI 3.27 to 5.94], *P* < 0.0001, *I*^2^ = 48%), 6-month (3.37, [95% CI 0.27 to 6.46], *P* = 0.03, *I*^2^ = 89%), and 12–60-month (3.59, [95% CI, 0.74 to 6.44], *P* = 0.01, *I*^2^ = 98%) follow-up. Sensitivity analysis was conducted in all subgroups. After removing each study from the analysis, no contrary result was found in 1-month, 3-month, and 12–60-month follow-up. However, when respectively excluding the data of Bartolucci, Chang, Chen, Perin, and Vrtoves, the significant effect of BMCs on LVEF in 6-month follow-up disappeared. Interestingly, after permanently removed the study of Martino, the positive effect retained in a sensitivity analysis. From the above analysis, whether BMC therapy improves LVEF at 6-month follow-up still need to be further discussed.

**Fig. 4:**
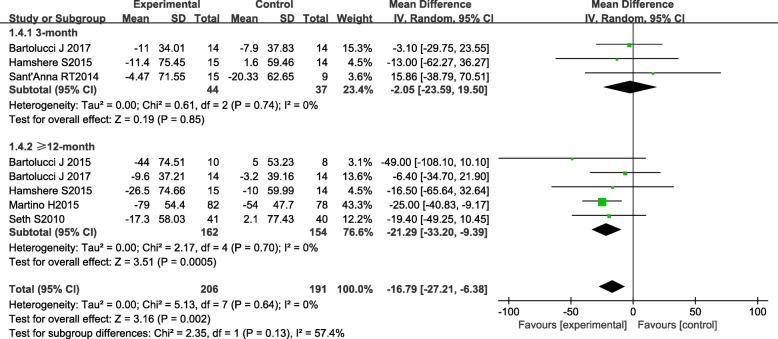
Forest plot of pooled left ventricular end-systolic volume (LVESV) with bone marrow-derived cell treatment compared with the control group

Six studies provided data of LVESV. The overall result showed a significant difference in LVESV between BMC group and controls, which was − 16.79 (95% CI − 27.21 to − 6.38, *P* = 0.002, *I*^2^ = 57.4%) (Fig. 4). Subgroup analysis revealed that BMC treatment significantly reduced LVESV in 12–60-month follow-up (− 21.29, [95% CI − 33.20 to − 9.39], *P* = 0.0005, *I*^2^ = 0%) compared with controls, whereas no significant effect was found in 3-month (− 2.05, [95% CI − 23.59 to 19.50], *P* = 0.85, *I*^2^ = 0%) follow-up period. The results of the sensitivity analysis were consistent with the original results.

**Fig. 5:**
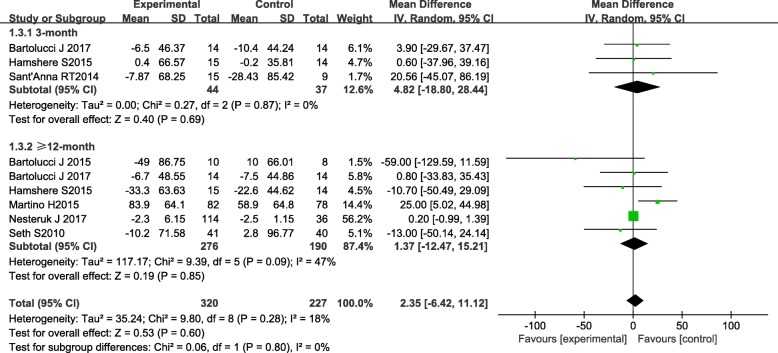
Forest plot of pooled left ventricular end-diastolic volume (LVEDV) with bone marrow-derived cell treatment compared with the control group

Data of LVEDV was measured in six trials. Results indicated that BMC therapy did not possess superior effect in improving LVEDV compared with the control group, with overall assessment (2.35, [95% CI − 6.42 to 11.12], *P* = 0.60, *I*^2^ = 18%), 3-month (4.82, [95% CI − 18.80 to 28.44], *P* = 0.40, *I*^2^ = 0%) and 12- to 60-month (1.37, [95% CI − 12.47 to 15.21], *P* = 0.85, *I*^2^ = 47%) follow-up (Fig. 5).

### Functional class, exercise capacity, and quality of life

BMC treatment significantly improved 6-min walk distance (53.16, [95% CI 25.17 to 81.10], *P* = 0.0002, *I*^2^ = 94%) and NYHA functional class (− 0.48, [95% CI − 0.65 to − 0.31], *P* < 0.0001, *I*^2^ = 45%) compared with controls, and the benefit of BMCs could be seen in all 3-month, 6-month, and 12- to 60-month follow-up period. For peak VO_2_, a significant difference was not detected between BMCs and control group (0.94, [95% CI − 3.15 to 5.02], *P* = 0.65, *I*^2^ = 67%) (Table 2). Only four studies measured this parameter [14, 19, 23, 35], subgroup analysis was not available, and the conclusion could not be drawn until more data were acquired.

**Table 2:** Pooled analysis of functional class, exercise capacity, quality of life, and clinical outcome

	No. of studies	Mean difference/risk ratio	95% CI	*P*
6-min walk distance				
Overall	9	53.13	25.47, 81.10	0.0002
3 months	6	47.92	7.95, 87.89	0.02
6 months	8	49.68	3.34, 96.01	0.04
≥ 12 months	6	63.91	0.06, 127.76	0.05
NYHA functional class				
Overall	6	− 0.48	− 0.65, − 0.31	< 0.0001
3 months	4	− 0.28	− 0.49, 0.08	0.007
6 months	4	− 0.52	− 0.96, − 0.07	0.02
≥ 12 months	6	− 0.63	− 0.83, − 0.43	< 0.0001
Peak VO_2_	4	0.94	− 3.15, 5.02	0.65
MlHFQ	4	− 18.41	− 29.90, − 6.92	0.002
MACE	6	0.79	0.60, 1.04	0.09
Rehospitalization	5	0.71	0.49, 1.04	0.08
All-cause mortality	17	0.74	0.56, 0.98	0.04
Cardiovascular death	8	0.65	0.29, 1.46	0.29

*NYHA* New York Heart Association, *MlHFQ* Minnesota Living with Heart Failure Questionnaire, *MACE* major adverse cardiovascular events

Patients’ quality of life was measured using the Minnesota Living with Heart Failure Questionnaire (MlHFQ). MlHFQ scores range between 0 and 105 with higher scores indicate a worst quality of life. The pooled outcome of 4 trials revealed that MlHFQ scores significantly decreased in BMC group compared with controls (− 18.41, [95% CI − 29.90 to − 6.92], *P* = 0.002, *I*^2^ = 0%) (Table 2).

### Clinical outcomes

Compared with controls, BMC therapy had a tendency to reduce the incidence of MACE and rehospitalization; however, the results did not reach statistical significance, with RR of 0.79 (95% CI 0.59 to 1.04, *P* = 0.09, *I*^2^ = 0%) and 0.71 (95% CI 0.49 to 1.04, *P* = 0.08, *I*^2^ = 0%), respectively (Table 2). Overall estimates showed a significant reduction in all-cause mortality in the BMC group compared with controls (0.74, [95% CI 0.56 to 0.98], *P* = 0.04, *I*^2^ = 0%). The same trend was observed in cardiovascular death analysis (Table 2).

### Subgroup analysis

Subgroup analysis stratifying studies based on the route of administration (intracoronary vs intramyocardial), cell types (BMNC vs BMSC), and dosage (10^6^ vs 10^8^) was performed with LVEF outcome owing to limited data. Comparison of administration route revealed that intracoronary injection of BMCs significantly improved LVEF in subgroups of 3, 6, and 12–60 months, while the effect of intramyocardial injection only manifested at 6-month follow-up (Table 3). For cell types, BMSC therapy appeared to have a superior effect than BMNC in improving LVEF throughout all follow-up period (Table 3). The different dosage might also affect the therapeutic efficacy of cell therapy. According to the subgroup analysis, patients treated with an injection of up to 10^8^ cells benefit more than patients received a lower cell dose in the improvement of LVEF at 3-month, 6-month, and 12–60-month follow-up (Table 3).

**Table 3:** Subgroup analysis of stem cell therapy in LVEF

Subgroup	Follow-up	No. of studies	Mean difference	95% CI	*P*
Injection route					
Intracoronary	3 months	6	4.96	3.42, 6.51	< 0.0001
	6 months	4	3.62	0.14, 7.11	0.04
	≥ 12 months	9	3.80	0.19, 7.40	0.04
Intramyocardial	3 months	2	2.24	− 3.25, 7.72	0.42
	6 months	4	5.29	1.94, 8.65	0.002
	≥ 12 months	3	1.15	− 1.30, 6.30	0.36
Cell type					
BMSC	3 months	6	4.58	3.74, 5.43	< 0.0001
	6 months	5	5.19	3.24, 7.04	< 0.0001
	≥ 12 months	5	5.04	1.00, 9.02	0.01
BMNC	3 months	4	3.77	2.25, 5.28	< 0.0001
	6-month	3	0.28	− 2.72, 3.28	0.85
	≥ 12 months	8	2.97	1.04, 4.89	0.002
Dosage					
10^6^	3 months	3	5.12	− 0.62, 10.86	0.08
	6 months	3	3.23	0.79, 5.68	0.009
	≥ 12 months	4	1.16	− 2.22, 4.54	0.50
10^8^	3 months	5	4.68	3.59, 5.77	< 0.0001
	6 months	3	6.23	4.62, 7.83	< 0.0001
	≥ 12 months	5	5.40	0.56, 6.64	0.0003

*BMNC* bone marrow mononuclear cells, *BMSC* bone mesenchymal stem cells, *LVEF* left ventricular ejection fraction

## Discussion

We believed that this is the largest meta-analysis on the subject of BMCs in treating CMP which included 20 studies with the most latest trials. The overall estimates of the meta-analysis demonstrated that BMC transplantation resulted in significant improvements in LVEF, LVESV, 6-min walk distance, NYHA functional class, MIHFQ scores, and all-cause mortality. Although significant differences were not observed in LVEDV, peak VO_2_, MACE, and rehospitalization rate, these results tended to in favor the BMC group, indicating that cell therapy was safe and feasible in patients with CMP without worsening the LV function and patients’ survival. However, a lot is still unknown or in a dispute about BMC therapy. Thus, several subgroup analyses were also conducted in an attempt to reveal its true effect.

Whether BMC therapy has a short-term or long-term effect is still the biggest argument. Subgroup analysis based on follow-up time showed that BMCs therapy had both short-term and long-term benefit on LVEF, 6-min walk distance, and NYHA functional class, whereas the effect on LVESV only emerged at 12–60-month follow-up. LVEF improved in short-term follow-up (3–6 month) without reduction of LVESV and LVEDV, suggesting that BMCs cause little changes in the remodeling process at the beginning and might exert its effect through a paracrine manner [37]. In long-term (12–60 month) period, the improvement of LVEF might be because of amelioration in LVESV, that is BMCs could contribute to the regeneration and differentiation of cardiomyocytes and as a consequence improve cardiac function by inhibiting LV remodeling [29, 38, 39]. The improvement in functional parameters at both short- and long-term follow-up indicated that BMC transplantation accelerated cardiac function recovery and the beneficial effects manifested soon after cell delivery and sustained.

Apart from functional markers, we also assessed clinical parameters such as MACE, rehospitalization, mortality rate, and patients’ quality of life. Results showed a trend towards a decrease in MACE and rehospitalization in BMC patients. Meanwhile, a significantly lower mortality rate and a better quality of life were detected in patients receiving stem cell therapy as compared with the controls. This suggests that the improvement in cardiac function after BMC treatment also translates into clinical benefits.

In stem cell therapy, intravenous administration, intracoronary infusion, and intramyocardial injection are the three currently available methods for cell delivery [40]. Yet, after decades of evaluation, no consensus has been reached on the optimal method of application. Some suggest that intramyocardial injection can exert better effect than intracoronary infusion because more cells are retained after intramyocardial injection (about 10% of total cells) compared with intracoronary infusion (about 3%) [41, 42]. Subgroup analysis in this meta-analysis indicated that patients with intracoronary infusion might benefit more than an intramyocardial injection, as patients treated with intracoronary infusion of BMCs demonstrated long-lasting benefit in LVEF whereas patients with intramyocardial injection exhibit improvement in LVEF only at 6-month analysis. However, given the fact that the sample size in the intramyocardial group was relatively small, this outcome needs to be interpreted with caution. Interestingly, at 6-month follow-up, the improvement of LVEF was greater in the intramyocardial group than the intracoronary group (5.29% vs 3.62%). Thus, the retention of more stem cells within the myocardium after injection might provide greater benefit to LV function as the previous investigation suggested. However, more clinical evidences are required until we can draw a definite conclusion.

Different cell types might also bring different outcomes. Unselected mononuclear bone marrow cells are the most commonly used seed cell in BMC therapy. Many consider that the potential beneficial effects might be attributed to the combined effects of all infused mononuclear cells rather than the small amount of progenitor cell or stem cell present in the bone marrow [43]. More recently, more and more investigators have focused on the effect of BMSC for CMP patients. It is suggested that BMSC possesses great potential for proliferation and can be cultured and amplified in vitro, t?>making them an ideal seed cell type for clinical cell therapy [44]. To provide more insights into the choice of cells, we conducted a subgroup analysis to compare the effect between BMNC and BMSC. Results showed that both cell types were effective in enhancing LVEF. At the same time, we noticed that BMSC produced more pronounced improvement in LVEF compared with BMNC data in all time groups, indicating that BMSC might be a more suitable seed cell for stem cell therapy.

Our analysis also included two studies using ixmyelocel-T in the treatment group. Ixmyelocel-T is an expanded multi-cellular therapy cultured from autologous BMNC. Previous studies have demonstrated that ixmyelocel-T therapy has the potential in decreasing secretion of proinflammatory cytokines after inflammatory stimuli and could efficiently remove apoptotic cells. This subpopulation of cells may have a potential role in tissue repair and regeneration [45–47]. The two studies enrolled in our meta-analysis, Henry et al. and Patel et al., concluded that although delivery of ixmyelocel-T in patients with ischemic dilated cardiomyopathy did not have a profound influence on LVEF, it resulted in a significant reduction in a major adverse cardiovascular event [21, 22]. This discovery might provide a new approach for cardiomyopathy treatment. Further studies should be conducted to evaluate the efficacy of this treatment option.

Poor cell engraftment and low survival of the transplanted cells are two major problems affecting the development of cell-based treatment [48]. Evidences show that most of the infusion cells die after a short amount of time, leaving only a small proportion that remains in the heart [49, 50]. Thus, clinicians hypothesized that the efficacy of cell-based therapy might improve in a dose-dependent manner. Several studies demonstrated that BMC therapy is associated with favorable effects on cardiac function, with a greater benefit observed with a higher cell dose [51–53]. Results of our subgroup analysis supported the previous finding, which found higher cell dose imparting greater benefit in the improvement of LVEF.

The scope of our results is limited by some limitations. The high degree of heterogeneity observed among the included studies is one of them. Although sensitivity and subgroup analyses were performed, we still could not completely eliminate the bias brought by different studies. Some of the factors should be taken into consideration, for example, baseline LVEF and BMC cell source. Baseline LVEF was proved to be an independent predictor of functional response to BMC therapy in previous investigation [36, 54]. However, limited data from the included studies hampered us to conduct a certain group of analysis. Furthermore, the comparison between autologous and allogeneic stem cell therapies in the treatment of heart disease is trending. Although autologous stem cells can avoid immunologic reaction during cell delivery, this type of cell is limited in immediate use due to the time-consuming nature of cell culturing and validation, which might miss the optimal window for treatment [55]. Allogeneic cell therapy is suggested to overcome the limitation of autologous cells, and already in clinical trials, allogeneic cells were found to have a superior effect on cardiac function compared with autologous cells without the sight of immunologic profile [56, 57]. Nevertheless, a study evaluating the effectiveness of autologous versus allogeneic cells is often without a control group. Therefore, analysis cannot be performed until more evidences from prospective randomized controlled trials are provided. These factors should be taken into consideration in future analysis.

## Conclusion

Overall, BMC therapy for CMP is safe and feasible without causing serious damage to the cardiac function and patients’ survival compared with standard treatment. Cell therapy results in improvement of LV function and amelioration of LV remodeling, and the benefit effect is durable. The enhancement of cardiac function leads to better patients’ survival and quality of life. Based on our subgroup analysis, intracoronary infusion of higher dosage (> 10^8^) of BMSC might bring about greater therapeutic efficacy. Future adequately powered trials with more reliable and more patient-centered outcomes are required to validate our result.

## Abbreviations

BMCs: Bone marrow-derived cells
BMNC: Bone marrow mononuclear cells
BMSC: Bone marrow mesenchymal stem cells
CMP: Cardiomyopathy
CMR: Cardiac magnetic resonance imaging
ECG: Electrocardiogram
ECHO: Echocardiography
LVEDV: Left ventricular end-diastolic volume
LVEF: Left ventricular ejection fraction
LVESV: Left ventricular end-systolic volume
MACE: Major adverse cardiovascular event
MlHFQ: Minnesota Living with Heart Failure Questionnaire
SPECT: Single-photon emission-computed tomography
WMD: Weighted mean difference
